# Effects of Epigallocatechin-3-Gallate on Matrix Metalloproteinases in Terms of Its Anticancer Activity

**DOI:** 10.3390/molecules28020525

**Published:** 2023-01-05

**Authors:** Hiroki Tanabe, Takuji Suzuki, Tomokazu Ohishi, Mamoru Isemura, Yoriyuki Nakamura, Keiko Unno

**Affiliations:** 1 Faculty of Health and Welfare Science, Nayoro City University, Nayoro 096-8641, Hokkaido, Japan; 2 Department of Food Science and Nutrition, Faculty of Human Life and Science, Doshisha Women’s College of Liberal Arts, Kyoto 602-0893, Japan; 3 Institute of Microbial Chemistry (BIKAKEN), Numazu, Microbial Chemistry Research Foundation, Numazu 410-0301, Shizuoka, Japan; 4 Institute of Microbial Chemistry (BIKAKEN), Laboratory of Oncology, Microbial Chemistry Research Foundation, Shinagawa, Tokyo 141-0021, Japan; 5 Tea Science Center, University of Shizuoka, Suruga-ku, Shizuoka 422-8526, Japan

**Keywords:** green tea, epigallocatechin-3-gallate, matrix metalloproteinases, cancer, transcription factors, reactive oxygen species

## Abstract

Epidemiological studies have shown that the consumption of green tea has beneficial effects against cancer. Basic studies have provided evidence that epigallocatechin gallate (EGCG) is a major contributor to these effects. Matrix metalloproteinases (MMPs) are zinc-dependent metalloproteinases with the ability to degrade the extracellular matrix proteins and are involved in various diseases including cancer in which MMPs have a critical role in invasion and metastasis. In this review, we discuss the effects of EGCG on several types of MMPs in the context of its anticancer activity. In the promoter region, MMPs have binding sites for at least one transcription factor of AP-1, Sp1, and NF-κB, and EGCG can downregulate these transcription factors through signaling pathways mediated by reactive oxygen species. EGCG can also decrease nuclear ERK, p38, heat shock protein-27 (Hsp27), and β-catenin levels, leading to suppression of MMPs’ expression. Other mechanisms by which EGCG inhibits MMPs include direct binding to MMPs to prevent their activation and downregulation of NF-κB to suppress the production of inflammatory cytokines such as TNFα and IL-1β. Findings from studies on EGCG presented here may be useful in the development of more effective anti-MMP agents, which would give beneficial effects on cancer and other diseases.

## 1. Introduction

**Figure 1:**
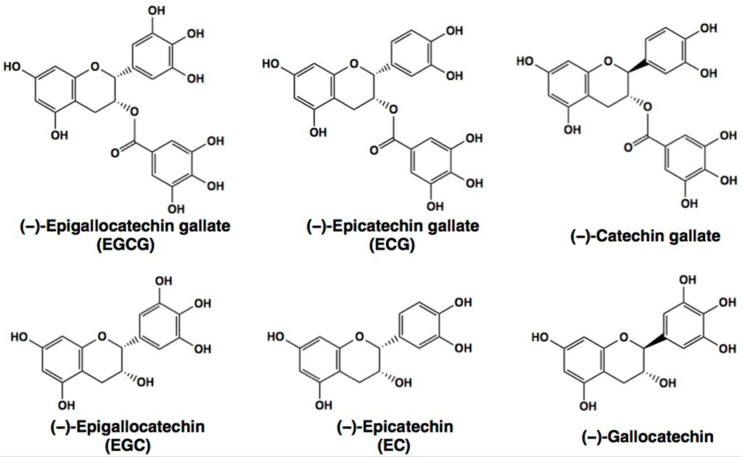
Chemical structures of (−)-epigallocatechin gallate (EGCG) and related catechins. ECG, (−)-epicatechin gallate; EGC, (−)-epigallocatechin, EC, (−)-Epicatechin.

Plant polyphenols are found in foods such as beans, tea, apples, onions, citrus fruits, broccoli, berries, grapes, and coffee and are believed to have health benefits including cancer prevention [1,2,3]. Green tea is a rich source of the flavanol catechins in which epigallocatechin gallate (EGCG) (Figure 1) is present most abundantly and a number of epidemiological evidence have shown that green tea intake has health-beneficial effects on cancer [1,2,3].

Animal and cell-based studies have supported these effects and proposed several mechanisms through which EGCG exerts anticancer effects. One of the most attractive ones is the reactive oxygen species (ROS)-mediated mechanism in which EGCG acts as strong antioxidants. Under certain conditions, EGCG can also act as a pro-oxidant. In our previous review articles, we discussed the anticancer mechanisms of EGCG and other dietary polyphenols via ROS-mediated pathways [4,5].

Matrix metalloproteinases (MMPs) are zinc-dependent metalloproteases capable of degrading extracellular matrix proteins [6,7,8]. In humans, at least 23 different MMPs have been found [8]. These MMPs exert a variety of biological activities and are involved in various diseases including cancer, cardiovascular diseases, diabetes, inflammation, and brain disorders [7,8,9].

Previous studies have examined the effects of EGCG on 10 MMPs, MMPs 1, 2, 3, 7, 8, 9, 11, 12, 13, and 14, and future studies will likely reveal the biological effects of EGCG on other MMPs. In this review, we discuss the effects of EGCG on these 10 MMPs in relation to its anticancer effects.

## 2. Anticancer Effects of EGCG

A large number of epidemiological studies have shown that the consumption of tea/green tea reduces various types of cancer [7]. For example, in a pooled analysis of eight population-based cohort studies, females who consumed green tea showed a decreased risk of total cancer mortality: pooled hazard ratios (HR) = 0.89 (95% confidence intervals (CI) = 0.83–0.96) for the consumption of 1–2 cups/day and HR = 0.91 (95% CI = 0.85–0.98) for the 3–4 cups/day [10]. For distal gastric cancer, the relative risk (RR) was 0.51 (95% CI = 0.30–0.86) in the highest category of green tea consumption (≥ 5 cups/day compared to < 1 cup/day) in women [11]. For endometrial cancer, the highest green tea consumption was associated with a reduced risk (RR = 0.78, 95% CI = 0.66–0.92), and an increase in green tea consumption of one cup per day was associated with an 11% decreased risk (RR = 0.89, 95% CI = 0.84–0.94) [12].

The anticancer effect of green tea intake has often been attributed to the effect of EGCG mainly based on animal and cell-based experimental results. For example, in an animal model of spontaneous hepatoma in C3H/HeNCrj mice, administration of 0.05% EGCG reduced the incidence of hepatoma-bearing mice by 67% compared to water-given control and the average number of hepatomas per mouse by 39% at week 65 [13]. In an intravesical tumor implantation model, the tumor-free incidence was 64% in rats given 200 μM EGCG but 0% in control rats [14].

EGCG has a dual function of antioxidant and pro-oxidant potential and EGCG-mediated ROS production and ROS-scavenging are considered to be responsible for its anticancer effects. EGCG promotes strong anticancer effects by multiple mechanisms including inhibition of nuclear factor-κB (NF-κB) signaling, inhibition of angiogenesis, promotion of apoptosis, and epigenetic modification by modulating DNA methylation and histone acetylation [15].

## 3. Modulation of MMPs by EGCG

MMPs are deeply associated with tumor growth, invasion, and metastasis. This has been demonstrated by a number of studies. For example, in a transplant model of human colon cancer, intraperitoneal injection of the MMP inhibitor BB-94 caused a 51% reduction in the tumor weight in nude mice compared to the control group [16]. The incidence of local and regional invasion was 35% in the BB-94 group, and 67% in the control group. In contrast to 33% of the control mice, only 10% in the BB-94 group had metastasis. The median survival times were 110 and 140 days in the control and BB-94 groups, respectively.

Transfection of rat tumor cells with the cDNA for tissue inhibitor of MMP (TIMP)2 caused a marked decrease in MMP activity [17]. Forced expression of this MMP inhibitor in four clones reduced tumor growth rate in vivo after subcutaneous injection and completely suppressed local tissue invasion. An MMP2-silencing experiment demonstrated that short hairpin (sh) RNA-transfected nasopharyngeal carcinoma CNE-1 cells showed inhibited cell colony formation compared to the control cells [18]. These findings indicate the pivotal roles of MMPs in cancer. It should be noted that tumor cells express MMPs and stromal cells such as fibroblasts are an important source of MMPs in tumors as well [19].

In a meta-analysis of 28 studies of breast cancer, Ren et al. [20] found that patients with high levels of serum MMPs had worse relapse-free survival (HR = 1.969, 95% CI = 1.460–2.655). HRs of MMP9 positivity with poor overall survival was 1.794 (95% CI = 1.330–2.420) by univariate analysis and 1.709 (95% CI = 1.157–2.526) by multivariate analysis. Therefore, clinical values of MMPs as prognostic biomarkers warrant future evaluation.

### 3.1. Roles of MMP1 (Collagenase 1) in Cancer

MMP1 has been described in various advanced cancers and correlated with poor survival of patients as shown in breast cancer and colorectal cancer [6,21]. Immunohistochemical studies on patients suggest that bone morphogenetic protein-6 (BMP-6) suppresses breast cancer metastasis [22]. BMP-6 inhibited the migration and invasion of breast cancer MDA-MB-231 cells, and this effect was attenuated by overexpression of MMP1. BMP-6 inhibited MMP1 promoter activity through the activator protein 1 (AP-1) response element, leading to downregulated MMP1 expression.

Cortez et al. [23] found that pro-inflammatory cytokine interleukin (IL)-17 stimulated MMP1 gene transcription and type I collagenase activity which was inhibited by MMP1 knockdown mediated by small interfering RNA (siRNA). IL-17 induced MMP1 in human cardiac fibroblasts by activating protein-38 (p38) mitogen-activated protein kinase (MAPK), extracellular signal-regulated kinase 1/2 (ERK1/2), AP-1, NF-κB, and CCAAT enhancer-binding protein beta (C/EBP-β). IL-17 may be a therapeutic target to reduce inflammation and injury. These findings suggest that inhibition of MMP1 may lead to anticancer effects.

Casimiro et al. [24] found that the receptor activator of the nuclear factor kappa ligand (RANKL) stimulated cell invasion of human breast cancer MDA-231BO2 cells through a type I collagen matrix. Upregulation of RANKL increased the levels of MMP1 through the activation of ERK/c-Fos and c-Jun aminoterminal kinase (JNK)/c-Jun pathways. siRNA-mediated knockdown of MMP1 of these cells attenuated their invasion and reduced bone metastasis was found in inoculated cells with MMP1 knockdown.

Wang et al. [25] showed that calmodulin-regulated spectrin-associated proteins promoted cell migration and invasion of colorectal cancer SW-620 cells through activation of the JNK/c-Jun/MMP-1 signaling pathway. Knockdown of MMP1 in these cells attenuated their invasion.

In breast cancer patients, expression of the POU class 1 homeobox 1 transcription factor (Pit-1) was positively correlated with the presence of both MMP1 and MMP13 [26]. Since distant metastasis is often observed in breast cancer patients with elevated expression of Pit-1, its downregulation may have a preventive effect on metastasis.

#### Effects of EGCG on MMP1

There have been several studies to demonstrate EGCG’s downregulation of MMP1. For example, EGCG at 50 μM downregulated collagen production and MMP1 in rat primary hepatic stellate cells (HSCs) and reduced the transcription of MMP1 in human HSC-derived TWNT4 cells [27]. EGCG suppressed the MMP1 expression induced by heat shock or tumor necrosis factor α (TNFα) in human fibroblasts [28,29]. In view of the involvement of MMP1 in the migration, invasion, and metastasis of, for example, colorectal cancer cells [25], EGCG’s inhibition of MMP1 may contribute to its anticancer effect.

Yun et al. [30] showed that EGCG downregulated TNFα-induced production of MMP1 and MMP3 in rheumatoid arthritis synovial fibroblast. EGCG also downregulated TNFα-induced phosphorylation of ERK1/2, p38, and JNK and inhibited the binding of AP-1 proteins to its response elements in synovial fibroblast.

EGCG suppressed collagen degradation in UV-B-exposed human dermal fibroblasts and inhibited UV-B-induced production of MMP1, MMP8, and MMP13 [31]. EGCG reduced UV-B-induced activation of ASK-1 and phosphorylation of MAPK, JNK, p38, and ERK1/2, in these cells.

### 3.2. Roles of MMP2 (or Gelatinase A) in Cancer

Analysis of tumor specimens from 12 patients with oral cavity squamous cell carcinoma suggested that MMP2 secreted from fibroblasts is involved in the invasion of oral cancer cells [32].

As described above, MMP2 knockdown significantly inhibited the colony formation and migration of nasopharyngeal cancer cells [18]. In esophageal carcinoma KYSE150 cells, siRNA against MMP2 caused inhibition of the mRNA and protein expression of MMP2, leading to reduced invasion and migration of these cells [33]. In a study using colorectal cancer HCT116 cells, the high invasiveness of these cells was shown to result from increased secretion of MMP2 and activation of focal adhesion kinase (FAK) signaling [34]. MMP2-knockdown with shRNA reduced cell migration. ARP-100, an MMP2 inhibitor, decreased the phosphorylation and protein levels of FAK signaling proteins, FAK, ERK, phosphorylated phosphatidylinositol-3-kinase (PI3K), and JNK, and reduced integrin-β1 and CD9 in the culture medium. The finding suggests that there is positive feedback in which MMP2 can activate FAK signaling leading to the upregulation of MMP2 itself in tumor cells.

Similarly, the results of siRNA experiments in lung cancer A549 cells suggested that MMP2 upregulates αVβ3 integrin mediated PI3K/protein kinase B (AKT) signaling to elevate vascular endothelial growth factor (VEGF) expression, resulting in enhanced angiogenesis [35]. Knockdown and overexpression experiments led Kesanakurti et al. [36] to show that MMP2 induces TNFα-mediated NF-κB activation and induces JNK-mediated apoptosis in glioma cells and xenograft cells.

Kargozaran et al. [37] found that human lung microvascular endothelial cells secreted MMP2 consistently, but human breast cancer MDA-MB-231 cells did only at a low level. When MMP2 expression and activity in the endothelial cells were inhibited by knockdown with siRNA or with an inhibitor OA-HY, respectively, the transmigration of the cancer cells across an endothelial monolayer barrier on Matrigel was abrogated. The finding suggests that the interaction between tumor cells and the vascular endothelium may have an important role in tumor invasion and metastasis.

Knockdown of MMP2 using MMP2 siRNA caused suppression of cell proliferation in glioma 4910 and 5310 cells established from mouse xenografts of human glioma tumors [38]. MMP2 downregulation also suppressed α5β1 integrin expression, MMP2/α5β1 interaction, the secreted levels of several cytokines including granulocyte-macrophage colony-stimulating factor (GM-CSF), IL-6, IL-8, IL-10, TNFα, VEGF, and platelet-derived growth factor (PDGF)-BB. The cells with MMP2-knockdown showed inhibited the binding activity of signal transducer and activator of transcription 3 (STAT3) to DNA. This inhibition was canceled by IL-6, suggesting suppression of IL-6/STAT3 signaling in the knockdown cells. The added recombinant MMP2 enhanced MMP2/α5β1 binding. The intracranial tumors of injected MMP2-knockdown cells showed much smaller in size, indicating a pivotal role of MMP2 in cancer.

Deng et al. [39] found that advanced glycation end products (AGEs) accelerate tumor invasion and metastasis, with upregulation of the receptor for AGEs (RAGE), specificity protein 1 (Sp1), and MMP2 protein expression and activity. AGEs caused an increase in pERK and MEK1/2 inhibitor-induced reduction of this phosphorylation led to suppression of the Sp1 expression, suggesting the involvement of the RAGE/ERK/Sp1/MMP2 pathway in AGE-induced tumor invasion and metastasis.

Microarray and quantitative reverse transcription-polymerase chain reaction (qPCR) analyses of hepatocellular carcinoma (HCC) HEP3B cells revealed that EGCG upregulated MMP1, MMP9, and long non-coding RNA ADAMTS9 and downregulated the expression of MMP11, MMP24 and a disintegrin and metalloprotease (ADAM) family protein ADAMTSL4 [40]. However, the contribution of these modulations of individual MMPs or in concert with EGCG’s anticancer effects remains to be determined.

#### Effects of EGCG on MMP2

**Figure 2:**
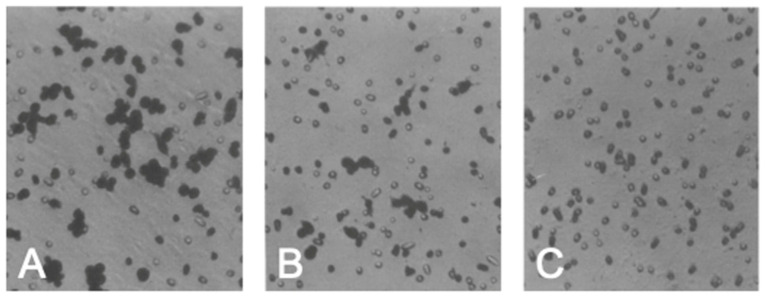
The number of the invaded cells stained with hematoxylin-eosin on the underside of the filter coated with Matrigel is reduced in the presence of green tea infusion in the cell incubation medium compared to its absence. (**A**), Invaded cells without green tea infusion; (**B**), Invaded cells with green tea infusion; (**C**), Chemotaxicell chamber without the added cells. Reproduced from [43]. Reprinted with permission from Ref. [43]. Copyright 1995 Elsevier.

Previous studies of our group showed that MMP2 and MMP9 were major MMPs secreted by murine tumor cells derived from Lewis lung cancer cells by gelatin zymography and affinity chromatography demonstrated that these MMPs were bound by EGCG immobilized on agarose gel [41]. EGCG inhibited the gelatinolytic activity of a mixture of MMP2 and MMP9 derived from these cells with an IC_50_ value of about 10 μM. (+)-Catechin and (−)-epicatechin (EC) up to 10 μM did not show activity. Oral administration of green tea and a catechin mixture rich in EGCG inhibited metastasis of LL2-L3 and melanoma B 16 cells, respectively, in the animal model [42,43]. When the invasion of LL2-L3 cells through an artificial basement membrane (Matrigel) was examined, green tea infusion reduced the number of the invaded cells (Figure 2). EGCG inhibited invasion of these cells with the IC_50_ value of about 25 μM, but EC did not [43].

**Figure 3:**
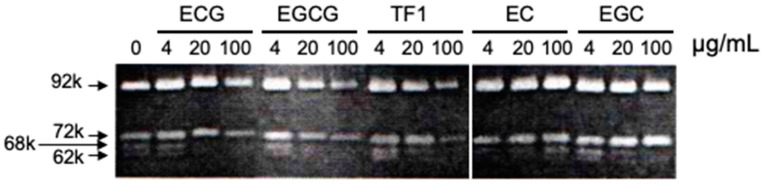
Gelatin zymography reveals that EGCG inhibited ProMMP2 (72k), activated MMP2 (68k and 62k), and MMP9 (92k). TF1, theaflavin-1. Reprinted with permission from Ref. [44]. Copyright 1999 American Chemical Society.

Maeda-Yamamoto et al. [44,45] demonstrated that in human fibrosarcoma HT1080 cells, EGCG suppressed the gelatinolytic activities ascribable to MMP2 and MMP9 caused by the downregulation of their mRNAs (Figure 3). EGCG also suppressed the expression of membrane-type1 MMP (MT1-MMP) mRNA. EGCG inhibited the phosphorylation of ERK1/2, which is required for MMP9 upregulation, and suppressed p38 activity, but not c-Jun. These findings suggest that suppression of ERK phosphorylation by EGCG is involved in the decreased expression of MMP2 and MMP9 mRNAs.

Dell’Aica et al. [46] found that EGCG directly inhibited MT1-MMP activity, leading to the accumulation of proMMP2 at the cell surface. Garbisa et al. [47] found that EGCG inhibited gelatinase activity of MMP2 and MMP9 and Matrigel invasion of human neuroblastoma SK-N-BE cells with a Ki value of 22 μM against MMP2 activity. EGCG was also shown to inhibit these MMPs in fibrosarcoma HT1018 cells independent of an excess of Zn^2+^ ions.

Bedoui et al. [48] found that in human aortic vascular smooth muscle cells (VSMCs), EGCG and ECG inhibited MT1-MMP activity. Thrombin-stimulation increased cell invasion which was inhibited by EGCG and EGC but not by EC. A ratio of MMP2/proMMP2 activity was decreased in thrombin-treated cells by EGCG or ECG. Decreased activity of MT1-MMP was also observed in these treated cells.

In breast cancer MCF-7 cells, EGCG decreased the activity of MMP2 and its protein and mRNA expression levels [49]. The expression of MMP9 was too low to be detected. EGCG also downregulated the expression of FAK, MT1-MMP, NF-κB, and VEGF and reduced cell adhesion. EGCG-induced reduction in phosphorylation of PI3K and ERK may be related to ECCG’s downregulation of MMP2.

In nasopharyngeal carcinoma TW01 and NA cells, EGCG inhibited cell proliferation, migration, and Matrigel invasion [50]. EGCG also reduced the phosphorylation of ERK and the nuclear levels of AP-1 and Sp1, leading to the downregulation of MMP2 and MMP9. EGCG inhibited the nuclear translocation of NF-κB and β-catenin along with the reduction of mRNA levels of epidermal growth factor receptor (EGFR). EGCG upregulated the expression of E-cadherin and β-catenin. EGCG caused inhibition of AKT phosphorylation to a lesser extent and no changes in the level of p38. The expression of MMP2 and MMP9 is regulated by several signaling pathways, including MAPK and PI3K/AKT signaling pathways affecting the downstream transcription factor AP-1 and Sp1 that modulates the promoter activity of MMP2 and MMP9 [51].

Annabi et al. [52] showed that EGCG inhibited MMP2 secretion in glioblastoma cells. Thus, EGCG exerts inhibitory effects on enzyme activity, gene expression and secretion of MMP2 from various tumor cells, resulting in reduced cancer cell invasion and metastasis.

Pramanik and Mishra [53] examined 127 post-operated samples from oral cancer patients. Results indicated that cancer tissues had increased protein and activity levels of MMP2 compared to adjacent normal samples. The MMP2 expression/activity was related to several signal-transduction pathways including ERK1/2 and Wingless and Int-1 (WNT)-β-catenin pathways and transcription factors such as NF-κB, AP-1, and Sp1. In Cal-27 and SCC4/9 cells derived oral squamous cell carcinoma, ECGC, and a MAPK-pathway inhibitor PD98059 diminished MMP2 activity and invasion/migration of these cells.

The effects of oral administration of EGCG on breast cancer patients undergoing treatment with radiotherapy were studied [54]. Results showed that EGCG caused reduced activation of MMP2 and MMP9 in patient sera along with lower serum levels of VEGF, and hepatocyte growth factor (HGF) compared to untreated patients.

In a xenograft model of human pancreatic cancer AsPC-1cells, EGCG inhibited cell proliferation, capillary tube formation, and migration of human umbilical vein endothelial cells (HUVECs), and these inhibitory effects were further enhanced in the presence of an ERK inhibitor [55]. EGCG decreased AsPC-1 xenograft tumor volume, angiogenesis, and metastasis together with downregulation of MMP2, MMP7, MMP9, and MMP12. Tumor samples from EGCG-treated mice had decreased ERK activity and enhanced p38 and JNK activities. However, it is not clear regarding the degree of contribution of the downregulation of individual MMPs to these effects.

In U87 glioblastoma cells, Djerir et al. [56] found that ConA-mediated MT1-MMP induction was inhibited by EGCG and catechin gallate, and endoplasmic reticulum stress biomarker GRP78 induction was inhibited by EGCG, catechin gallate, and gallocatechin gallate, whereas proMMP2 activation was inhibited by EGCG and gallocatechin gallate. Surface plasmon resonance study showed that gallated catechins interacted better than their ungallated analogs with MT1-MMP as well as with MT1-MMP binding partners such as MMP2 and TIMP2, suggesting the importance of the galloyl moiety in EGCG’s inhibition of MT1-MMP-mediated proMMP2 activation.

Prostate-specific antigen (PSA) is a serine-protease and can degrade extracellular matrix proteins, thereby affecting cell migration and metastasis. Pezzato et al. [57] found PSA could degrade gelatin and Matrigel components. PSA also degraded proMMP2 resulting in the generation of active MMP2, which was inhibited by EGCG. PSA’s activation of proMMP9 was not observed.

Li et al. [58] found that EGCG induced apoptosis in glioma U251 cells via the 67 kDa laminin receptor (67LR) because 67LR knockdown reduced apoptosis. EGCG abrogated the invasion and proliferation of these cells. The MAPK pathway was found to be involved in EGCG’s effects. EGCG also caused decreases in the mRNA levels of MMP2 and MMP9. EGCG upregulated P38 and JNK. Although pERK1/2 was upregulated at 25 μg/mL EGCG, the higher concentrations of EGCG decreased the level of pERK1/2. Based on the previous findings in a toxoplasma-induced inflammation model of astroglia showing that MG132, an NF-κB inhibitor, reduced expression of pNF-κB, MMP2, and MMP9 and ERK inhibitor PD98059 mitigated pERK1/2, pNF-κB, MMP2, and MMP9 expression [59], it is plausible to consider that 67LR, an EGCG sensing molecule [60], is the molecule responsible for EGCG’s downregulation of signaling of ERK1/2-NF-κB-MMP9/MMP2.

Additional examples of studies in which EGCG’s modulation of MMP2 and MMP9 was demonstrated are listed in Table 1.

### 3.3. Roles of MMP3 in Cancer

MMP3 (or stromelysin 1) has a role in tumor invasion and metastasis [72]. MMP3 exhibits a number of activities to promote tumor development. In addition to degrading various ECM components, MMP3 can activate MMP9 and the collagenases, and degrade a number of cell surface molecules, including E-cadherin which contributes to cancer development [73].

In malignant mesothelioma SPC212 cells, zymography with fibronectin showed that MMP3 activity was enhanced when treated with various factors such as epidermal growth factor (EGF), transforming growth factor-α (TGF-α), HGF, insulin-like growth factor (IGF)-II, and basic fibroblast growth factor (bFGF) [9].

Lochter et al. [74] examined MMP3′s regulation of ductal morphogenesis, apoptosis, and neoplastic progression in mammary epithelial cells by generating cells expressing an inducible autoactivating transgene. Inducer-triggered MMP3 expression caused cleavage of E-cadherin, phenotypic changes such as the disappearance of E-cadherin and β-catenin at cell–cell contact sites, upregulation of MMPs including MMP9, and resulted in showing the contribution of MMP3 to invasive nature of cells expressing MMP3.

In a later discussion, Sternlicht et al. [73] proposed a mechanism in which MMP3 modulates genes containing lymphoid enhancer factor-binding sites such as those of myc, cyclin-D1, E-cadherin and MMP3, 7, 9, and 13. Kwon et al. [75] showed that filamin A interacting protein 1-like, an inhibitor of cell migration and invasion, reduces β-catenin levels, leading to the transcriptional downregulation of WNT target genes such as MMP3 and MMP9, resulting in inhibition of metastasis. Thus, MMP3 inhibition can be expected to have anticancer effects.

#### Effects of EGCG on MMP3

**Figure 4:**
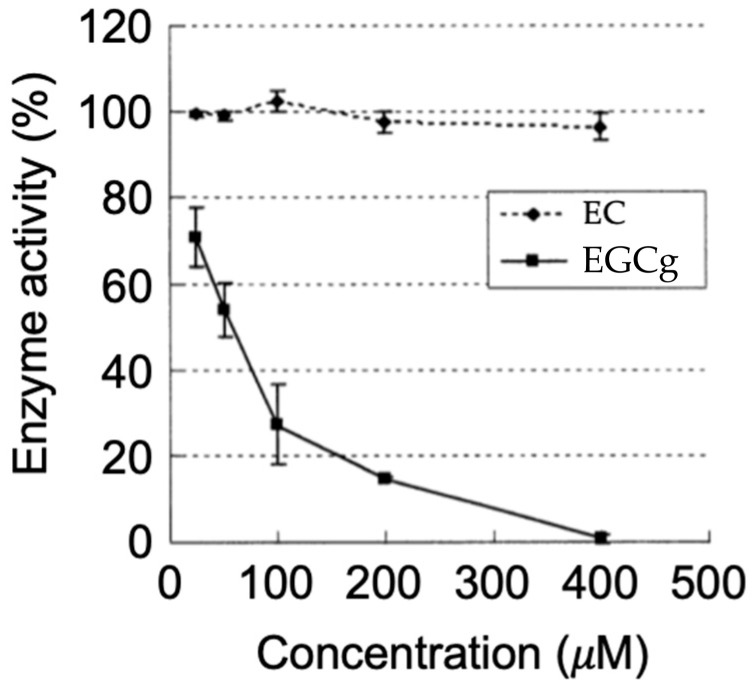
Dose-dependent inhibition of MMP3 activity by EGCG. EGCG (EGCg) inhibits MMP3 activity in lung cancer LL2-Lu3 cells, but EC did not. Reprinted with permission from Ref. [76]. Copyright 2000 Wiley-Blackwell.

A previous study of our group showed that EGCG inhibited MMP3 activity with an IC_50_ value of ca. 50 μM in the culture medium of mouse Lewis lung carcinoma-derived cells (Figure 4) [76]. Lee et al. [77] found that water extracts of green, white, and black teas reduced UVB-induced skin damage in a photoaged hairless mouse model together with the reduced expression of MMP3.

Downregulation by EGCG of TNFα-induced production of MMP1 and MMP3 in rheumatoid arthritis synovial fibroblast [30] is described above.

### 3.4. Roles of MMP7 (Matrilysin) in Cancer

MMP7 (matrilysin-1) plays important roles in the growth, invasion, and metastasis of tumors [78]. Unlike many MMPs, MMP7 is typically expressed in epithelial cells, and MMP7 expression increases at the invasive front in esophageal adenocarcinoma which may be partly attributable to the activation of PI3K [79]. Secreted MMP7 may modify the tumor microenvironment by stimulating stromal cell migration and invasion.

Although in Min/+ mice, MMP7 mRNA was detected in 88% of Min adenomas and localized to epithelial-derived tumor cells [80], MMP7-deficient mice with a targeted disruption of the gene had a 60% reduction in mean tumor multiplicity and decreased average tumor diameter. The findings suggest that MMP7 may act as a suppressor of the Min phenotype in a capacity independent of matrix degradation.

#### Effects of EGCG on MMP7

As described above, EGCG downregulated MMPs including MMP7 in AsPC-1 xenograft tumors, but MMP’s contribution to EGCG’s effects is to be determined [55].

In contrast, in human colorectal cancer HT-29 cells, EGCG (10–100 μM) increased both intracellular and extracellular proMMP7 protein levels with a significant upregulation of mRNA expression [78]. EGCG also activated ERK1/2, JNK, and p38. Induction of proMMP7 expression by EGCG was also shown in another human colorectal adenocarcinoma cell line, Caco-2.

These conflicting results should be examined in detail in future investigations.

### 3.5. Roles of MMP8 in Cancer

Cao et al. [81] showed that MMP8 secreted by irradiated liver nonparenchymal cells enhanced the migration and invasion of HCC through modulation of AMP-activated protein kinase (AMPK)/mammalian target of rapamycin signaling, providing a possible mechanism in sublethal irradiation-induced HCC metastasis observed often in radiotherapy.

MMP8 may be prognostic for certain cancers. When liver metastasis samples from 419 colorectal cancer patients were examined for cancer-associated markers including serum MMP8, the pre- and postoperative high levels of MMP8 were associated with worse 10-year overall survival [82]. In contrast, tumor MMP8 expression may be a favorable prognosis in pancreatic ductal adenocarcinoma [83]. Thus, further studies would be required for the significance of MMP8 in prognosis.

#### Effects of EGCG on MMP8

EGCG’s inhibition of UV-B-induced production of MMP1, MMP8, and MMP13 [31] is described above.

### 3.6. Roles of MMP9 (Gelatinase B) in Cancer

In oral squamous cell carcinoma (OSCC), knockdown of MMP9 lead to inhibition of cell migration, proliferation, interactions between endothelial cells, tumor growth of nude mouse xenografts, angiogenesis, and OSCC cell metastasis to mouse lymph nodes [84]. Knockdown of MMP9 decreased the expression of RhoC, Src, and F-actin.

Ding et al. [85] found that TNFα markedly increased MMP9 expression and decreased collagen IV expression in hCMEC/D3 cells and the effect was attenuated by pretreatment with 2,6-diisopropylphenol, an intravenous anesthetic agent. MMP9 knockdown with siRNA resulted in the inhibition of TNFα-induced downregulation of collagen IV. Experiments using the inhibitors revealed that TNFα upregulated MMP9 expression through activation of Ca^2+^/calmodulin-dependent protein kinase II (CAMK II)/ERK/NF-κB signaling pathway.

In fibrosarcoma HT1080 cells, MMP9 knockdown caused increased cell adhesion and inhibited tumor cell migration. In a nude mouse xenograft model, MMP9 knockdown inhibited tumor growth, reduced tumor volume, and prolonged survival time [86].

#### Effects of EGCG on MMP9

Several studies of the effects of EGCG on MMP9 are included in Table 1 because these studies examined MMP2 and MMP9 at the same time.

Sato and Seiki [87] analyzed the 5′-flanking sequence of MMP9 and found that three motifs, homologous to the binding sites for AP-1, NF-κB, and Sp1 proteins, contributed to induction by TPA and TNFα. Comparison of the promoter structures of TPA-inducible MMP1 and MMP3 revealed that the signal to the AP-1 sites is common but the signals to the NF-κB or Sp1 sites in MMP9 are the unique determinant for MMP9 induction because these sites are not present in MMP1 and MMP3 promoters.

In lung carcinoma 95-D cells, EGCG suppressed their invasion, downregulated the expression of MMP9, and reduced the nuclear localization of NF-κB [88]. EGCG also reduced intracellular oxidative stress, which may contribute to its suppression of tumor invasion via the downregulation of MMP9 and NF-κB.

Sen et al. [89] studied the effect of EGCG on MMP9 in human breast cancer MDA-MB-231 cells. Results indicated that EGCG repressed the activity, protein, and mRNA expression of MMP9 and increased the expression of TIMP1. EGCG abrogated the activation of FAK and ERK, reduced the adhesion to fibronectin and vitronectin, and reduced the mRNA expression of the integrins α5β1 and αVβ3. In addition, EGCG suppressed NF-κB expression and DNA binding activity of NF-κB and activator AP-1 to the MMP9 promotors, indicating EGCG causes transcriptional deregulation of the MMP9 gene in these cells.

The nicotine in cigarette smoke has been correlated to tumor propagation. Khoi et al. [90] investigated the effects of EGCG on nicotine-induced cell invasion and MMP9 activity in human endothelial ECV304 cells. Results showed that EGCG abrogated the MMP9 expression and transcriptional activity. EGCG inhibited nicotine-suppressed production of ROS and nicotine-induced NF-κB and AP-1 activation. Experiments with an expression of mutated NF-κB and AP-1 decoy demonstrated that NF-κB and AP-1 are involved in the nicotine-stimulated MMP9 expression. These findings suggest that MMP9 is under the control of ROS, NF-κB, and AP-1.

In cervical cancer HeLa cells, EGCG caused growth inhibition and cell death through apoptosis [91]. EGCG also inhibited the invasion and migration of these cells, suppressed the gene expression of MMP9, and increased the gene expression of TIMP1.

In colon cancer HCT116 cells with wild-type p53 and HT-29 cells with mutant p53, EGCG induced apoptosis in a p53-independent manner [92]. EGCG suppressed the expression levels of VEGF and MMP9 regardless of the p53 status. Compound C, the AMPK inhibitor, attenuated these effects in HT-29 cells, suggesting that AMPK is involved in the downregulated expression of VEGF and MMP9 in EGCG-treated cells.

The Tax oncogene is expressed in HTLV-1-infected cells. Harakeh et al. [93] found that EGCG exerted cytotoxicity in HTLV-1 positive ATL HuT-102 and C91-PL cells and reduced expression of Tax. EGCG decreased NF-κB activity in these cells and also the mRNA and protein levels and activity of MMP9.

In bladder cancer SW780 cells, EGCG inhibited cell proliferation, migration, and invasion [94]. EGCG induced apoptosis in these cells by activation of caspases-8, -9, and -3, Bax, and poly-ADP ribose polymerase. In mice bearing SW780 tumors, EGCG injection resulted in decreases in tumor volume and weight. EGCG downregulated the expression of NF-κB and MMP9 in both protein and mRNA levels in the tumor and SW780 cells. The NF-κB inhibitor SC75741 canceled the inhibitory effects of EGCG on the proliferation and migration of SW780 cells.

When human monocytic THP-1 cells were induced with phorbol myristate acetate (PMA) to differentiate into macrophages, EGCG inhibited the expression of MMP9 and activation of ERK1/2, p38, and JNK [95]. Anti-67LR antibody attenuated the EGCG’s inhibition on the expression of MMP9 and activation of ERK1/2, p38, and JNK. Additionally, the EGCG’s inhibition of ERK1/2 phosphorylation was abrogated in 67LR-ablated cells. As mentioned above, 67LR may be involved in the mechanism of EGCG’s downregulation of MMP9 via the ERK1/2-NF-κB-MMP9 signaling pathway.

### 3.7. Roles of MMP13 in Cancer

As described above, expression of the POU class 1 homeobox 1 transcription factor (Pit-1) in breast cancer patients was positively correlated with the presence of both MMP1 and MMP13 [26]. In SCID mice, knockdown of MMP13 blocked lung metastasis of injected Pit-1-overexpressing MCF-7 cells.

MMP13 is expressed in the mesenchymal stromal cells of giant cell tumors of bone [96]. IL-1β and TNFα upregulated MMP13 at mRNA and protein levels. Inhibition of the ERK and JNK signaling pathways inhibited the upregulation of MMP13 in these cells. Knockdown of transcription factor Runx2 resulted in the downregulation of MMP13, suggesting that Runx2 may involve cytokine mediated MMP13 expression in these cells.

High-mobility group box-containing protein 1 (HBP1) is a transcription factor to function as a tumor suppressor in various cancers. Analysis of oral tumor specimens showed that the low HBP1/high MMP13 status was associated with metastatic potential [97]. MMP13 knockdown significantly reduced invasion enhanced by HBP1 siRNA, suggesting a role of MMP13 in cancer invasion and metastasis.

#### Effects of EGCG on MMP13

RNA-seq and qPCR analyses of HCC HEP3B cells revealed that EGCG’s modulation of various MMPs including upregulation of MMP13 as mentioned above [40].

Chiang et al. [98] found an increase in MMP13 mRNA and protein expression in OSCC cells relative to normal oral keratinocytes. Esophageal squamous cell carcinoma also showed high MMP13 mRNA expression. Treatment with >5 μM EGCG suppressed the expression and activity of MMP13 in oral cancer OEC-M1 cells, suggesting an anticancer effect of EGCG through the downregulation of MMP13.

### 3.8. Roles of MT1-MMP (MMP14) in Cancer

Membrane type-1 matrix metalloproteinase (MT1-MMP) is a transmembrane MMP that triggers intracellular signaling and regulates extracellular matrix degradation associated with tumor angiogenesis and inflammation [56]. One of the most important roles of MT1-MMP seems to activate MMP2 [56,99].

In human breast cancer MDA-MB-231 cells, transgenic knockdown of MT1-MMP reduced invasiveness and response to invasion stimulus of HGF [100]. In clinical breast cancer tissues, immunohistochemistry detected both membranous and cytoplasmic localization of MT1-MMP and showed stronger staining of tumor cells compared to normal mammary epithelial cells. The transcript levels in tumor tissues were insignificantly higher compared to normal tissues and the significantly higher levels in tumors were detected in patients with shorter disease-free 10 years from breast cancer-related causes.

Nguyen et al. [101] demonstrated that forced MT1-MMP expression in low-invasive LNCaP prostate cancer cells enhanced ROS activity and MT1-MMP knockdown in DU145 cells decreased ROS activity. Overexpression of MT1-MMP increased oxidative DNA damage in these cells and the ROS scavenger N-acetylcysteine blocked the MT1-MMP-mediated increase in cell migration and invasion. Experiments with cells expressing MT1-MMP mutant cDNAs revealed the requirement of the proteolytic activity of cell surface MT1-MMP for ROS activation, suggesting a role of MT1-MMP proteolytic activity in the induction of invasive phenotypes via ROS activation.

#### Effects of EGCG on MT1-MMP

As mentioned above, in U87 glioblastoma cells, EGCG inhibited ConA-mediated MT1-MMP induction and proMMP2 activation which is likely to be caused by the binding interaction of EGCG with MT1-MMP and MMP2 [56].

Yamakawa et al. [102] found that EGCG downregulated MT1-MMP, which promotes endothelial cell migration and tube formation and inhibited the invasion of HUVECs at 10 μM. EGCG suppressed tube formation by HUVECs and angiogenesis in vivo in a dorsal air sac model. EGCG administration resulted in suppressed tumor growth of colon 26 NL17 and Meth A sarcoma in mice with suppressed angiogenesis.

In U87 glioblastoma cells, chemotactic migration induced by TGF-β was suppressed by silencing either MT1-MMP or epithelial-mesenchymal transition-related protein SNAIL together with the reduced phosphorylation of decapentaplegic homolog (Smad)2/3 and STAT3 [103]. Similar results were observed by pharmacological inhibitors of STAT3 including EGCG, suggesting that EGCG’s suppression of MT1-MMT could lead to reduced migration of these cells.

## 4. Mechanistic Consideration

There are several mechanisms by which EGCG regulates MMPs. These include: (i) inhibition of gene expression of MMPs; (ii) inhibition of activation of proMMPs to active MMPs; (iii) direct inhibition of the enzymatic activity of MMPs; (iv) downregulation of TIMP: (v) downregulation of cell surface receptors of growth factors; and (vi) modulation of release of cytokines from cancer cells.

### 4.1. Inhibition of Gene Expression of MMPs

**Figure 5:**
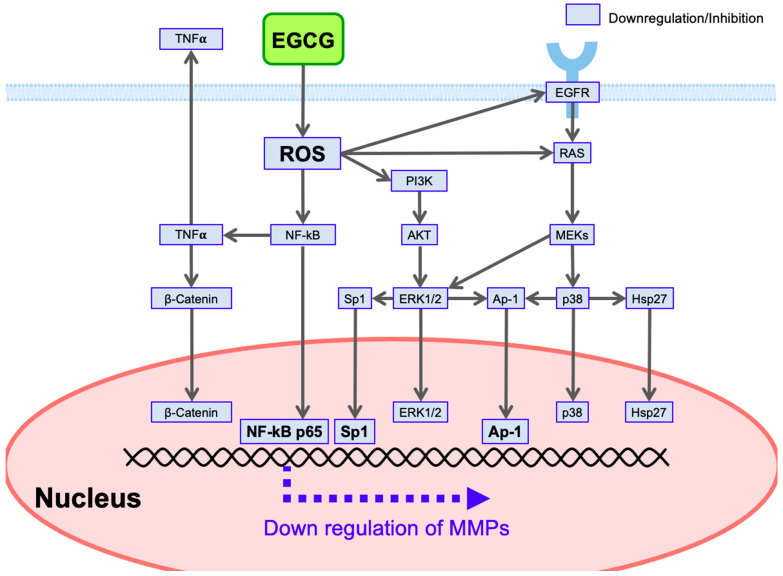
EGCG-mediated downregulation of MMPs via ROS-triggered signal transduction pathways.

EGCG has anticancer effects including the downregulation of MMPs. We have postulated polyphenol-triggered ROS-mediated anticancer pathways as one of the most attractive mechanisms. This scheme shows that, for example, ROS-scavenging properties of EGCG would lead to downregulation of NF-κB, resulting in downregulation ofMMP9 gene expression in view of the presence of the binding site of NF-κB p65 in the promoter (Table 2, Figure 5). Based on previous and additional information [29,104,105,106], we present a possible mechanism to explain how EGCG downregulates various kinds of MMPs (Figure 5). Table 2 lists the selected transcription factor-binding sites present in the promotors of MMPs [107,108].

Other studies also found that EGCG downregulates NF-κB: EGCG inhibits the nuclear translocation of NF-κB and β-catenin [50]. Several studies described above showed modulation of AP-1 by EGCG. For example, EGCG reduces the nuclear levels of AP-1 and Sp1 in nasopharyngeal carcinoma cells [50]. As for Sp1, EGCG suppresses the expression, DNA binding activity, and transactivation activity of Sp1 protein in LNCaP prostate cancer cells [109].

In addition to these canonical transcriptional factors, there is a possibility that several member molecules associated with the ROS-triggered signal transduction pathways are involved in the nuclear modulation of MMP expression. These include β-catenin [73,110], ERK [110], p38 [110], heat shock protein-27 (Hsp27) [111], and STAT [110] (Figure 5). Phosphorylases are expected to phosphorylate target proteins to involve in the modulation of transcriptional activity on MMPs. Nuclear Hsp27 may interact with Sp1, leading to the upregulation of MMP2 [111,112]. However, future studies are required to reveal the role of these molecules in the modulation of MMP gene expression.

Several conflicting results have been reported as exemplified by the EGCG’s effect on p38 [30,45,50,78,113]. The reasons for these differences may include those in cell types, EGCG concentrations, culture incubation time, and cell densities used in experiments. Future studies will be needed to understand the EGCG’s effects on signaling molecules.

### 4.2. Inhibition of Activation of proMMPs to Active MMPs

Several studies to show EGCG’s inhibition of activation of proMMPs to active MMPs are described above.

### 4.3. Direct Inhibition of Enzymatic Activity of MMPs

**Figure 6:**
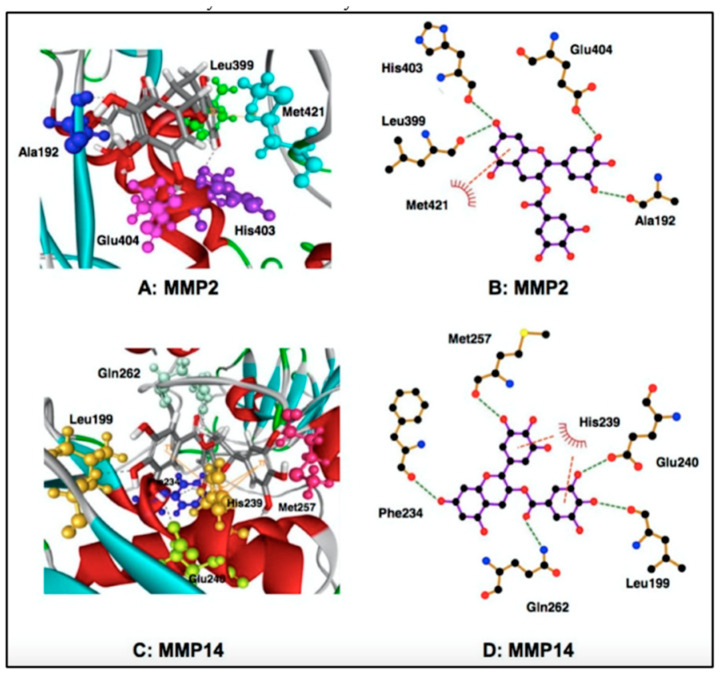
Molecular docking analyses for binding interaction of EGCG with MMP2 and MT-1 MMP (MMP14). Reprinted with permission from Ref. [99]. Copyright 2017 Springer Netherlands.

The results of affinity chromatography using EGCG immobilized on agarose showed the direct binding of EGCG with MMP2 and MMP9. Inhibition of proMMP2/active MMP2 activities was found for EGCG and ECG, but not EC and EGC [44,99] (Figure 6). Computational molecular docking analyses showed EGCG’s binding to MMP2 and MT1-MMP (Figure 6). The results showed that in the MMP2-EGCG interaction the 15 amino acid residues have interaction energy of >2 kcal/mol, and the A192, L399, H403, E404, M421 amino acid residues are located close to the EGCG binding site [99,114]. The P423 residue can engage in the complex formation with EC and EGC, but not with EGCG, and this binding interaction may be a reason why EC and EGC cannot inhibit MMP2 activity.

MT1-MMP activates proMMP2 to form MMP2. According to molecular docking analyses, MT1-MMP can strongly interact with both EGCG and ECG, and the galloyl group is responsible for the interaction [99,114]. In this interaction, the E240, L199, Q262, M257, F234, and H239 residues are involved and the H239 residue is considered to be involved in the interaction between EGCG and MT1-MMP [99].

### 4.4. Upregulation of TIMPs

EGCG upregulates TIMPs, leading to the inhibition of MMPs.

### 4.5. Modulation of Signaling from Cell Surface Receptors

Various kinds of growth factors promote tumor growth and invasion. For example, EGF produced in cancer-associated macrophages gives such effects on tumor cells [115]. As shown in Figure 5, EGCG downregulates EGFR [50], leading to suppression of EGF’s effect.

EGCG binds to the cell surface receptor 67LR and inhibits signal pathways involving ERK1/2 and p38 [60,95], which would lead to downregulation of the MMPs’ promoter activity (Figure 5).

In AGE-mediated tumor invasion and metastasis may be suppressed by EGCG’s inhibition of the RAGE/ERK/Sp1/MMP2 pathway [39].

### 4.6. Reduction of Circulating Cytokines

Inflammatory cytokines such as TNFα and IL-1β produced by tumor cells may promote MMP expression in stromal and epithelial cells [116,117,118]. As shown in Figure 5, EGCG’s downregulation of NF-κB would prevent the production of these cytokines as exemplified by TNFα (Figure 5) in tumor cells [105], leading to disruption of tumor and stromal interactions.

### 4.7. Other Mechanisms

As described above, Kwon et al. [34] suggested positive feedback in which MMP2 can activate FAK signaling leading to the upregulation of MMP2 itself in tumor cells. One can expect that EGCG’s binding with MMP2 would block this feedback loop. Sternlicht et al. [73] proposed MMP3 as a natural promoter that cleaves cell surface E-cadherin to release β-catenin, leading to increased transcription of carcinogenic genes such as c-myc gene. EGCG’s inhibition of MMP3 would be helpful to prevent cancer development.

## 5. Closing Remarks

Expressions of MMPs are different depending on cell types and stimulants. For example, in human fibrosarcoma HT-1080 and HCC SK-Hep-1, cells express both MMP2 and MMP9 [119], and glioblastoma T-98G cells express MMP2 but MMP9 only after stimulation with PMA. Uterine leiomyosarcoma SK-UT-1 cells express no MMPs but PMA induces MMP9.

Human melanoma A-2058 cells express MMP2 but MMP9 at a low level [120]. PMA at 100 ng/mL gave no effect on MMP2 secretion but potently upregulated MMP9 secretion. TNFα gave no significant effect on the expression of MMP2 but increased MMP9 secretion. IL-1β had little effect on MMP2 or MMP9 secretion, but IL-1β at 25 ng/mL reduced MMP2 level and MMP9 secretion. Lipopolysaccharide (LPS) had no significant effect on MMP2 secretion but enhanced MMP9 secretion was found up to 25 µg/mL. Therefore, extraordinarily complex mechanisms must be operating which can cause inconsistent results and conclusions. Nevertheless, future studies on the modulation of MMPs by EGCG would provide useful information on the beneficial effects of green tea intake on diseases including cancer.

## Figures and Tables

**Table 1:** Modulation of MMP2 and MMP9 by EGCG in selected papers.

Human Cell Types	Major Findings on Effects of EGCG (↓): Inhibition/Downregulation; (↑): Activation/Upregulation; (±): No Effect	References
Fibrosarcoma HT-1080 cells	Gelatinolytic activity (IC_50_ = 20 μM) (↓), Matrigel invasion (IC_50_ = less than 0.1 μM) (↓).	Garbisa et al. [61]
Kaposi’s sarcoma IMM cells	Cell growth (↓), endothelial cell growth (↓), invasion (↓), apoptosis (↑) (at high concentration). Gelatinolytic activity in endothelial cell supernatants (↓), formation of new capillary-like structures (↓). In xenografted mice: tumor growth (↓), angiogenesis (↓).	Fassina et al. [62]
Tumor bronchial epithelial cells	Cell migration (↓), MMP2 mRNA and protein expression (↓), MT1-MMP (±). MMP9 expression was not detected.	Hazgui et al. [63]
Oral squamous cell carcinoma-9 cells	Cell migration (↓), motility (↓), adhesion (↓), p-FAK (↓), p-Src (↓), snail-1 (↓), vimentin (↓), urokinase-type plasminogen activator (↓), EMT (↓), PMA-induced invasion (↓), PMA-induced MMP9 expression (↓). In xenografted mice: tumor growth (↓).	Chen et al. [64]
Neuroblastoma both SK-N-BE2 and SH-SY5Y cells	Matrigel invasion (↓), MMP2 (↓), MMP9 (↓), pAKT (↓), NF-κB (↓), VEGF (↓), bFGF (↓), Notch-1 (↓), hTERT (↓), PCNA (↓), E-cadherin (↑), Caspase 8 (↑), Bid (↑), Bax (↑), Bcl-2 (↓), Caspase 3 (↑), ICAD (↑). Survivin blocked these effects of EGCG.	Hossain et al. [65]
A431 and SCC13 skin cancer cells	Cell death (↑), MMP2 (↓), MMP9 (↓), phosphorylation of β-catenin (↑), nuclear β-catenin (↓), casein kinase1α (↑), phosphorylation of glycogen synthase kinase-3β (↓).	Singh and Katiyar [66]
Uveal melanoma M17 cells	Cell migration (↓), secreted MMP2 activity (↓), mRNA and protein expression (±), expressions of MMP2 (↓), TIMP2 (↑), RECK (↑), pERK1/2 (↓), p38 and JNK levels (±).	Chang et al. [67]
Nasopharyngeal carcinoma TW01 cells	Proliferation (↓), migration (↓), invasive (↓), MMP2 (↓), MMP9 (↓), E-cadherin (↑), β-catenin (↑), pERK (↓), AP-1 (↓), Sp1 (↓). In xenografted mice: tumor growth (↓), p53 (↑), p21 (↑), apoptosis (↑) caspase 3 (↑), nuclear translocation of NF-κB (↓), β-catenin (↓).	Fang et al. [50]
Doxorubicin-sensitive human breast cancer MCF7 cells	MMP2 activity (↓), MMP9 activity (↓). These activities were not detected in Doxorubicin-sensitive MCF7 cells.	Nowakowska et al. [68]
Cholangiocarcinoma HuCC-T1 cells	Cell viability (↓), growth (↓), invasion (↓); MMP2/MMP9 activity (↓), apoptosis (↑), Bax/Bcl-2 (↑), Caspase 3/9 (↑), mutant p53 (↓). In xenografted mice: tumor growth (↓),MMP2/9 (↓), Notch-1(↓), PCNA (↓)	Kwak et al. [69]
BE(2)-C neuroblastoma cells	Cell viability (↓), RXRα (±), RXRβγ (±), RXRγ (↑) Bcl-2 (±), cleaved PARP (↑), MMP2 mRNA (↓) MMP2 activity (↓), MMP2 protein (±), MMP9 mRNA (±), MMP9 activity (↓).	Farabegoli et al. [70]
ES-2 ovarian cancer cells	TGF-β-induced MMP2 (↓), TGF-β-induced EMT biomarkers (fibronectin, Snail, Slug, Smad-3 phosphorylation) (↓). EC gave no effects on TGF-β-induced MMP2.	Sicard et al. [71]

EMT, epithelial-mesenchymal-transition; Bcl-2, B-cell CLL/lymphoma 2; ICAD, inhibitor of caspase-activated DNase; PARP, poly-ADP ribose polymerase; PCNA, proliferating cell nuclear antigen; RECK, reversion-inducing-cysteine-rich protein with kazal motifs; RXR, retinoid X receptor.

**Table 2:** Transcription factor-binding sites in human MMP promotors.

Human MMPs	Transcription Factors *
MMP1 (Collagenase 1)	AP-1
MMP2 (Gelatinase A)	AP-2, Sp1
MMP3 (Stromelysin-1)	AP-1
MMP7 (Matrilysin)	AP-1
MMP9 (Gelatinase B)	AP-1, Sp1, NF-κB (p65)
MMP10 (Stromelysin-2)	AP-1
MMP12 (Metalloelastase)	AP-1

* Transcription factors related to this review are listed selectively.

## Data Availability

Not applicable.

## Abbreviations

67LR	67 kDa laminin receptor
ADAM	A disintegrin and metalloprotease
ADAMTS9	ADAM metallopeptidase with thrombospondin type 1 motif 9
ADAMTSL4	ADAMTS like 4
AGEs	Advanced glycation end products
AKT	Protein kinase B
AMPK	AMP-activated protein kinase
AP-1	Activator protein 1
BMP-6	Bone morphogenetic protein-6
CAMK II	Calmodulin-dependent protein kinase II
CI	Confidence interval
EC	Epicatechin
ECG	Epicatechin gallate
EGCG	Epigallocatechin gallate
EGF	Epidermal growth factor
EGFR	Epidermal growth factor receptor
ERK1/2	Extracellular signal-regulated kinase 1/2
F-actin	Filamentous actin
FAK	Focal adhesion kinase
GM-CSF	Granulocyte-macrophage colony-stimulating factor
HCC	Hepatocellular carcinoma
Hsp27	Heat shock protein 27
HUVECs	Human umbilical vein endothelial cells
IGF-II	Insulin-like growth factor-II
IL	Interleukin
JNK	c-Jun aminoterminal kinase
LPS	Lipopolysaccharide
MAPK	Mitogen-activated protein kinase
MMPs	Matrix metalloproteinases
MT1-MMP	Membrane-type1 MMP
NF-κB	Nuclear factor-κB
ORs	Odds ratios
OSCC	Oral squamous cell carcinoma
p38	Protein-38
PDGF	Platelet-derived growth factor
PI3K	Phosphatidylinositol-3-kinase
Pit-1	POU class 1 homeobox 1 transcription factor
PMA	Phorbol myristate acetate
PSA	Prostate-specific antigen
qPCR	Quantitative reverse transcription-polymerase chain reaction
RAGE	Receptor for AGEs
RANKL	Receptor activator of the nuclear factor kappa ligand
RhoC	Ras homolog family member C
ROS	Reactive oxygen species
SiRNA	Small interfering RNA
Sp1	Specificity protein 1
STAT3	Signal transducer and activator of transcription 3
TGF-β	Transforming growth factor-β
TIMP	Tissue inhibitor of MMP
TNFα	Tumor necrosis factor α
VEGF	Vascular endothelial growth factor
WNT	Wingless and Int-1
